# Women’s Quality-of-Life Trajectories During Pregnancy and Postpartum Periods by Mode of Birth: A Prospective Cohort Study

**DOI:** 10.1007/s10995-026-04298-5

**Published:** 2026-08-25

**Authors:** Alayna Carrandi, Yanan Hu, Maria Forslund, Jenny Gamble, Debra Creedy, Emily Callander, Valerie Slavin

**Affiliations:** 1 Monash Centre for Health Research and Implementation, Monash University, Clayton, VIC Australia; 2 Department of Obstetrics and Gynecology, Institute of Clinical Sciences, Sahlgrenska Academy, University of Gothenburg, Gothenburg, Sweden; 3 School of Nursing and Midwifery, Monash University, Peninsula Campus, Frankston, VIC Australia; 4 School of Nursing and Midwifery, Griffith University, Queensland, Australia; 5 School of Public Health, University of Technology Sydney, Sydney, Australia; 6 Women, Newborn and Children’s Services, Gold Coast University Hospital, Southport, QLD Australia

**Keywords:** Maternity care, Value-based health care, Health-related quality of life, Pregnant women, Postpartum women

## Abstract

**Objectives:**

To determine the quality-of-life scores of women during pregnancy and postpartum and compare the differences by mode of birth.

**Methods:**

A secondary analysis of a prospective, observational cohort study comprising 2866 pregnant women in Queensland, Australia. Women’s quality-of-life scores were collected at 36 weeks of gestation and 6- and 12-week postpartum and converted into utility values.

**Results:**

On average, quality-of-life utility scores increased from 36 weeks of gestation to 12 weeks postpartum, regardless of birth type. After adjusting for socioeconomic characteristics, women who went on to have a planned caesarean section had a significantly lower utility score at 36 weeks of gestation compared to women who went on to have a spontaneous vaginal birth (adjusted mean difference =  − 0.07; 95% CI − 0.11, − 0.02); women who did not have select birth type had a statistically significantly lower utility score at 36 weeks of gestation compared to women who had a select birth type (adjusted mean difference =  − 0.05; 95% CI − 0.08, − 0.02). No significant differences were observed for other birth types at 6- and 12-week postpartum.

**Conclusions for Practice:**

Women’s quality of life improves from late pregnancy to postpartum. Further research is needed to develop targeted interventions to improve quality of life among women at higher risk of low quality of life and adverse pregnancy and birth outcomes, however we did not identify any differences by mode of birth.

## Significance

What Is Already Known on This Subject?

What This Study Adds?

Previous studies reported on changes in women’s quality of life from pregnancy to postpartum used subscale scores (physical and mental) only.

A secondary analysis of a prospective, observational cohort study comprising 2866 pregnant women in Queensland, Australia. Women’s quality-of-life scores were collected at 36 weeks of gestation and 6- and 12-week postpartum and converted into utility values.

## Objectives

Quality-of-life assessments have become increasingly recognised among researchers, clinicians, policymakers, and healthcare administrators for their ability to optimise the provision of care by prioritising investment in areas that result in improvement of quality of life. The concept of quality of life encompasses a wide range of personal and social facets that affect a person’s physical health and social and psychological functioning (The Whoqol Group, 1998). Quality-of-life assessments are performed using psychometrically validated instruments (Hennessy et al., 1994; Wu et al., 2021) and measuring quality of life before and after delivery of health care allows for comparison of the outcomes of care across services and interventions. Quality-of-life assessments can also support clinical decision-making at the point of care. These assessments can be used to evaluate intervention effects by quantifying how the receipt of care impacts the quality of life. Such effect measures are then often used in combination with cost data to determine the cost-effectiveness of interventions and inform the efficient allocation of resources that maximises improvements in quality of life at the least cost (Alzboon & Vural, 2019; Hennessy et al., 1994). Accordingly, quality-of-life assessments play a fundamental role in the development and implementation of evidence-based practices in health policy, planning and practice.

Exploring the interplay of healthcare outcomes and maternal quality of life has become popular in understanding how maternity care might influence quality of life (Wang et al., 2013). Pregnancy and childbirth are major life events that can substantially affect women’s physical, psychological, and social well-being. Throughout pregnancy, a woman’s body undergoes significant physiological and anatomical changes. Even in healthy and uncomplicated pregnancies, these normal changes and their interactions with biopsychosocial factors, can affect a woman’s quality of life (Alzboon & Vural, 2019; Lagadec et al., 2018). For instance, factors such as educational status, socioeconomic status, parity, maternal age, and gestational age at birth are known to influence quality of life during pregnancy and maternal health outcomes (Ishaq et al., 2022; Lagadec et al., 2018; Mazúchová et al., 2018; Park & Choi, 2018). The lowest mean quality-of-life scores among pregnant women are commonly reported in the third trimester of pregnancy and the highest mean scores in the second trimester (Alzboon & Vural, 2019; Vachkova et al., 2013; Wang et al., 2013; Wu et al., 2021). A decline in quality of life throughout pregnancy occurs regardless of the type of pregnancy—whether normal, simple pathological or complex pathological (Morin et al., 2019). This is unsurprising since low quality of life is associated with negative physical, psychological, and psychosocial experiences that may increase as pregnancy progresses (Lagadec et al., 2018). Based on prior research, quality of life is known to be impacted by pregnancy-related physical symptoms such as fatigue, back pain, pelvic pain and insomnia; psychological discomforts, including diminished body image and sexual life, fear of birth, concerns for the child’s and existing children’s health and worries about spousal relationship changes, employment and financial issues; and psychosocial experiences, such as domestic family violence, inadequate social support, previous sexual or physical abuse, alcohol and other drugs, intergenerational trauma and inadequate housing or finances (Alzboon & Vural, 2019; Bai et al., 2018; Vachkova et al., 2013; Wang et al., 2013; Wu et al., 2021). The culmination of negative physical, psychological, and psychosocial experiences may consequently affect a woman’s usual activities and birth outcomes, and further reinforce low quality of life.

Mode of birth may further contribute to these experiences, as different birth types are associated with varying levels of physical recovery, obstetric complications, psychological outcomes, and maternal functioning. For example, caesarean birth has been associated with increased postoperative pain and delayed recovery, whereas instrumental vaginal birth may be linked to pelvic floor trauma and psychological distress (Martínez-Galiano et al., 2019; Mousavi et al., 2013; Nolens et al., 2018; Torkan et al., 2009). However, previous studies examining maternal outcomes by mode of birth have often focused primarily on isolated physical or mental health domains rather than broader patient-reported quality of life measures (Alzboon & Vural, 2019; Wang et al., 2013).

This study aimed to determine the quality-of-life scores (including overall physical health, mental health, social health, pain, fatigue, and overall perceived quality of life) of women during pregnancy and postpartum and compare the differences among birth types. We hypothesised that quality-of-life scores would differ according to mode of birth.

## Methods

This is a secondary analysis of a prospective, observational cohort study comprising 2866 pregnant women in one metropolitan tertiary hospital (Gold Coast University Hospital) in Queensland, Australia. Consecutive pregnant women were recruited into the study over a 14-month period (January 2021–March 2022) when booking to birth at the hospital. Pregnant women were included if they were 18 years or older, had sufficient ability to communicate in English and had mobile and/or email access. Reflecting primary study aims, women with significant pre-existing urogynaecology disorders were also excluded.

Recruitment used a waiver of consent process to contact women. All women were informed about the study during pregnancy by a Participant Information Sheet and waiver of consent statement. Women were informed that a research midwife would contact them by phone at 36 weeks to discuss the research project and were offered the ability to opt out by text message. A research midwife (VS) screened and contacted all eligible women by phone. Consenting women were sent the first survey link by text message. Surveys were subsequently automated. Non-responding women were sent two text message follow-up reminders.

Ethical approval to conduct this study was obtained from Gold Coast Hospital and Health Service Human Research Ethics Committee (HREC/2020/QGC/67333) and Griffith University (GU Ref No: 2020/957). Written informed consent to participate was obtained from all participants.

### Data Collection and Analysis

Analyses were performed using SAS 9.4. Women’s demographic data (marital status, ethnicity, and education) and health-related quality of life were collected at 36 weeks of gestation and 6- and 12-week postpartum using three online surveys. Other demographics (age, parity, pre-pregnancy body mass index, and postal code) and birth outcomes (mode of birth and weeks of gestation at birth) were collected using routinely collected electronic hospital records. We categorised mothers’ socioeconomic status based on the postcode of usual residence using the index of relative socio-economic disadvantage (IRSD) in the socio-economic indexes for areas (SEIFA) 2016—a classification system developed by the Australian Bureau of Statistics that ranks geographic areas across Australia (Census of Population and Housing: Socio-Economic Indexes for Areas (SEIFA), 2016). Lower IRSD scores indicate greater disadvantage, whereas higher scores indicate lower levels of disadvantage. The IRSD is designed to capture disadvantage only and does not provide a measure of advantage. Areas with low IRSD scores may be characterised by a higher prevalence of low-income households, low levels of education, low rental costs, unemployment, and jobless families. Descriptive analysis was conducted for maternal sociodemographic characteristics and mode of birth. We identified a select group of women who had composite birth type—a term birth (37–42 weeks) with spontaneous onset, vertex positioning of the baby, and had no episiotomy and no epidural.

Women’s health-related quality of life was collected via the Patient-Reported Outcomes Measurement Information System Global-10 (PROMIS-10) short form questionnaire (Hays et al., 2009), which has been validated for use in a sample population of pregnant and postpartum women (Slavin et al., 2019). The PROMIS-10 questionnaire comprises 10 items that assess general domains of health and functioning, including overall physical health, mental health, social health, pain, fatigue, and overall perceived quality of life. The health states were converted into utility values by mapping to the EQ-5D (Thompson et al., 2017). Utility values vary between 0 and 1 (1 being the best possible quality of life).

Crude mean quality-of-life scores were reported for the total study population, by mode of birth, and for the select birth type, for all women who completed the PROMIS-10 at each time point. Generalised linear models with a gamma distribution and log link function were used to model quality of life at each time point, adjusting for marital status, educational status, socioeconomic status, parity, age, pre-pregnancy body mass index and country of birth. These variables were chosen because they are known to influence quality of life during pregnancy (Ishaq et al., 2022; Lagadec et al., 2018; Mazúchová et al., 2018; Park & Choi, 2018). Adjusted mean quality-of-life utility scores and adjusted mean differences were calculated by mode of birth and for the select birth type using the least square method. Statistical significance was reported at a level of 0.05. Given the presence of missing data across the three time points, analyses were conducted separately for each survey round. For each time point, complete-case analysis was applied, excluding individuals with missing data on variables included in the model. As a result, the sample size differed across survey rounds. No imputation procedures were undertaken.

## Results

Of the 2866 women included in this study, a majority had completed postsecondary education (59.59%) and 7.92% were in the most disadvantaged socioeconomic category (SEIFA quartile 1; 7.92%) (Table 1). Most pregnant women in this study were nulliparous (57.96%), of normal weight (47.07%), married (77.42%) and non-Indigenous (55.58%). The average age of participants at birth was 30.63 years old. Around half of all women (52.51%) had spontaneous vaginal birth. Approximately one-third of the women (31.51%) had caesarean section and 9.49% had assisted vaginal birth. Just over half of all caesarean Sects. (56.59%) were unplanned. 974 (33.98%) women had our composite birth type (a term birth (37–42 weeks) with spontaneous onset, vertex positioning of the baby, and had no episiotomy and no epidural).

**Table 1:** Sociodemographic characteristics and mode of birth of the sample population

Characteristic	Sample population	
Total, N (%)	2866	(100.00)
Age at birth		
Age in years, mean (SD)	30.63	(5.04)
Education completed		
Less than Year 12, N (%)	197	(6.87)
Secondary school Year 12, N (%)	457	(15.95)
Apprenticeship or diploma, N (%)	651	(22.71)
University/tertiary degree, N (%)	1057	(36.88)
Missing, N (%)	504	(17.59)
Socioeconomic advantage (measured via SEIFA)		
1 *(the most disadvantaged)*, N (%)	227	(7.92)
2, N (%)	455	(15.88)
3, N (%)	1211	(42.25)
4 *(the least disadvantaged)*, N (%)	677	(23.62)
Missing, N (%)	296	(10.33)
Parity		
Parous, N (%)	1045	(36.46)
Nulliparous, N (%)	1661	(57.96)
*Missing, N (%)*	160	(5.58)
Pre-pregnancy body mass index (kg/m^2^)		
Underweight (< 18.5), N (%)	113	(3.94)
Normal weight (18.5–24.9), N (%)	1349	(47.07)
Overweight (25.0–29.9), N (%)	690	(24.08)
Obese (≥ 30.0), N (%)	529	(18.46)
*Missing*, N (%)	185	(6.45)
Marital status*		
Single, N (%)	142	(4.95)
Married/de facto, N (%)	2219	(77.42)
*Missing*, N (%)	505	(17.62)
Ethnicity		
Australian, N (%)	1593	(55.58)
Indigenous Australian, N (%)	104	(3.63)
Asian, N (%)	62	(2.16)
New Zealand, N (%)	171	(5.97)
Other, N (%)	427	(14.90)
*Missing*, N (%)	509	(17.76)
Mode of birth		
Spontaneous vaginal birth, N (%)	1505	(52.51)
Assisted vaginal birth, N (%)	272	(9.49)
Forceps, N (%)	93	(3.24)
Vacuum, N (%)	179	(6.25)
Caesarean section, N (%)	903	(31.51)
Planned caesarean section, N (%)	392	(13.68)
Unplanned caesarean section, N (%)	511	(17.83)
*Missing, N (%)*	185	(6.45)
Select birth type—term, spontaneous onset, vertex, no episiotomy, no epidural		
Yes, N (%)	974	(33.98%)
No, N (%)	1568	(54.71%)
*Missing*, N (%)	324	(11.30%)

Married/de facto includes partnered but not living together

*SEIFA* socio-economic indexes for areas, *SD* standard deviation

^*^Single includes divorced/separated and widowed

**Fig. 1:**
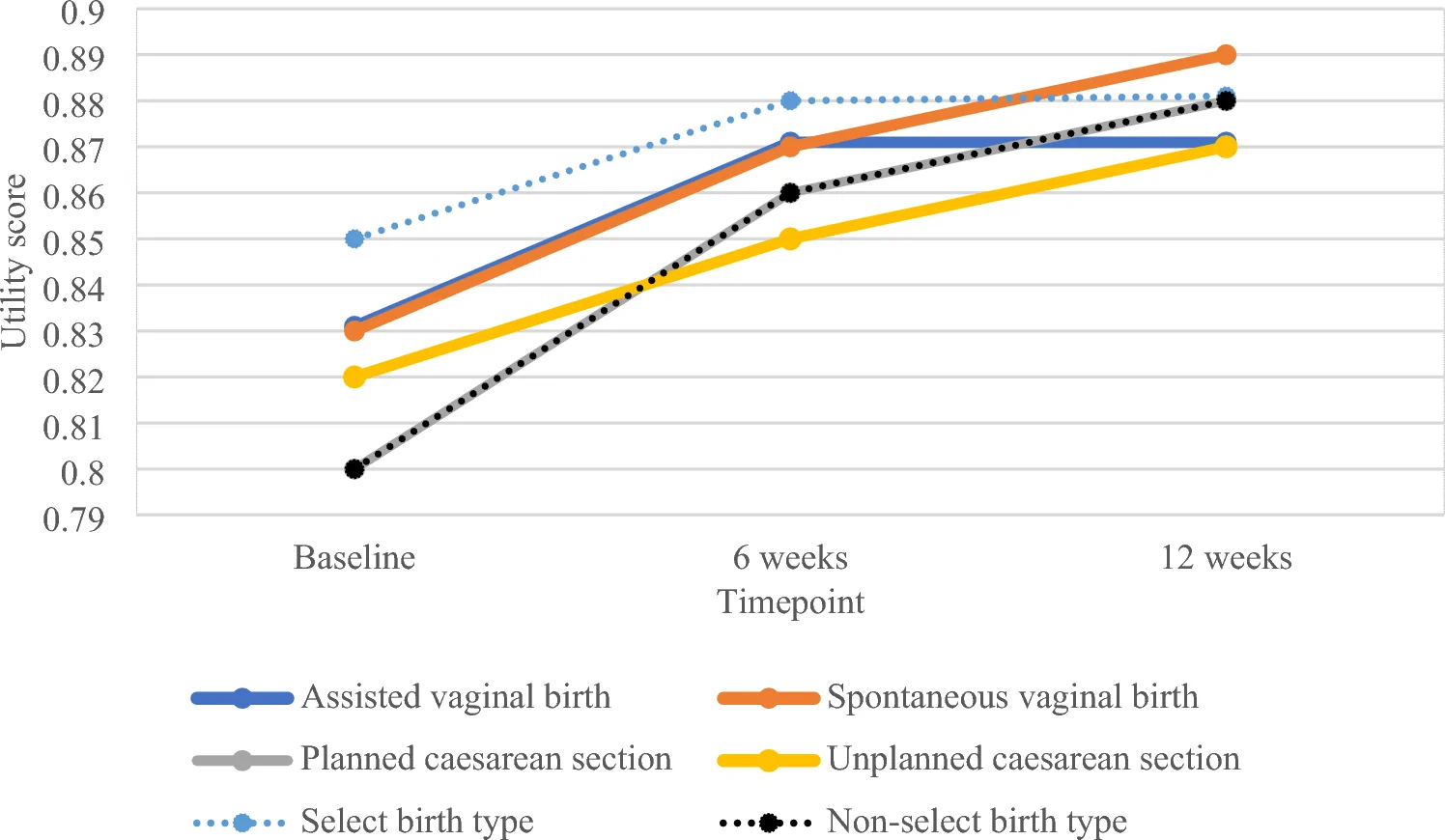
Mean quality-of-life utility score over time, by mode of birth

On average, quality-of-life utility scores increased from late pregnancy to 12 weeks postpartum (Fig. 1). Based upon unadjusted scores, Women who had a caesarean section (either planned or unplanned) had a lower crude utility score at 36 weeks of gestation and 6 weeks postpartum compared to those who did not. Women who had the composite birth type had higher utility score at 36 weeks of gestation and 6 weeks postpartum compared to those who did not. Quality-of-life utility scores remained static for women who had an assisted vaginal birth from 6 to 12 weeks postpartum and women who had an assisted vaginal birth or unplanned caesarean section had lower mean quality-of-life utility scores at 12 weeks postpartum compared to women who had a spontaneous vaginal birth. Appendix 1 presents the mean quality-of-life utility scores and sample sizes at each time point.

After adjusting for sociodemographic confounders, women who had a planned caesarean section had a statistically significantly lower quality-of-life utility score prior to birth, at 36 weeks of gestation, compared to women who had a spontaneous vaginal birth (adjusted mean difference =  − 0.07; 95% CI − 0.11, − 0.02) (Table 2). Women who did not have the composite birth type had a statistically significantly lower quality-of-life utility score at 36 weeks of gestation compared to women who had the composite birth type (adjusted mean difference =  − 0.05; 95% CI − 0.08, − 0.02) (Table 2). No significant differences were observed between spontaneous vaginal birth and other birth types at 6- and 12-week postpartum. Appendix 2 presents the coefficients across sociodemographic cohorts in the generalised linear model.

**Table 2:** Adjusted mean quality-of-life utility score at 36 weeks of gestation, 6 weeks postpartum and 12 weeks postpartum, by mode of birth

	Pregnancy		Postpartum			
	36 weeks of gestation		6 weeks postpartum		12 weeks postpartum	
	Adjusted mean (95% CI)	aMD (95% CI)	Adjusted mean (95% CI)	aMD (95% CI)	Adjusted mean (95% CI)	aMD (95% CI)
Mode of birth						
Spontaneous vaginal birth N = 326	0.81 (0.75, 0.87)	Reference	0.87 (0.82, 0.93)	Reference	0.89 (0.84, 0.94)	Reference
Assisted vaginal birth N = 55	0.81 (0.75, 0.88)	0.01 (−0.04, 0.06)	0.87 (0.81, 0.94)	− 0.0009 (− 0.05, 0.05)	0.89 (0.83, 0.95)	− 0.001 (− 0.04, 0.04)
Planned caesarean section N = 84	0.76 (0.70, 0.81)	− 0.07 (− 0.11, − 0.02)	0.87 (0.82, 0.93)	− 0.0001 (− 0.04, 0.04)	0.88 (0.83, 0.93)	− 0.01 (− 0.05, 0.02)
Unplanned caesarean section N = 101	0.79 (0.74, 0.86)	− 0.02 (−0.05, 0.02)	0.86 (0.80, 0.92)	− 0.02 (− 0.05, 0.02)	0.88 (0.83, 0.93)	− 0.02 (− 0.05, 0.02)
Select birth type—term, spontaneous onset, vertex, no episiotomy, no epidural						
Yes N = 241	0.82 (0.77, 0.88)	Reference	0.88 (0.83, 0.94)	Reference	0.90 (0.85, 0.95)	Reference
No N = 325	0.78 (0.73, 0.83)	− 0.05 (− 0.08, − 0.02)	0.87 (0.81, 0.92)	− 0.02 (− 0.05, 0.01)	0.88 (0.84, 0.93)	− 0.02 (− 0.04, 0.01)

## Conclusions for Practice

This study presented mean quality-of-life utility score trajectories and compared the differences by mode of birth at 36 weeks of gestation, 6 weeks postpartum and 12 weeks postpartum. On average, quality-of-life scores increased from 36 weeks of gestation to 12 weeks postpartum. Women who had a planned caesarean section had a significantly lower quality-of-life score prior to birth, at 36 weeks of gestation compared to women who had a spontaneous vaginal birth, after adjusting for marital status, educational status, socioeconomic status, parity, age, and body mass index. Similar lower quality-of-life scores have been found prior to birth at 36 weeks of gestation for women who did not have a composite measure of birth type (term, spontaneous onset, vertex, no episiotomy, no epidural), compared to women who had a select birth type. No differences in quality of life between women who had a spontaneous vaginal birth and all other birth types were observed after the time of birth, at 6 or 12 weeks postpartum. Although statistically significant differences were observed, the clinical relevance of these differences should be interpreted cautiously. Previous literature has shown that minimally important differences for EQ-5D scores vary according to baseline health status, population characteristics, and clinical context, suggesting that a single uniform threshold may not be appropriate. Given the generally high baseline health status observed in our populations, even relatively small utility differences may still be relevant.

Our study did not find a statistically significant relationship between mode of birth and quality of life during the postpartum period, while other research exploring the influence of mode of birth on quality of life has shown mixed results. Our findings were aligned with a cross-sectional study (n = 1375) that found no statistically significant differences in quality of life between postpartum women who had a caesarean section and spontaneous vaginal birth (Huang et al., 2012). Other previous research has found more favourable quality-of-life subscale scores for postpartum women who had a spontaneous or assisted vaginal birth, compared to women who had a caesarean section, specifically regarding physical functioning (Martínez-Galiano et al., 2019; Mousavi et al., 2013; Nolens et al., 2018; Torkan et al., 2009). This could be because of the high prevalence of wounds and pain following caesarean section (Jin et al., 2016) and the effect this may have on a woman’s physical functioning in the postpartum period. Our study also found that quality-of-life scores for women who had an assisted vaginal birth remained static from 6 to 12 weeks postpartum and these women had lower quality-of-life scores at 12 weeks compared to women who had a planned caesarean section or spontaneous vaginal birth. This could reflect ongoing pain or incontinence often associated with episiotomy and perineal trauma (Gün et al., 2016).

Low quality of life during pregnancy may affect pregnancy and birth outcomes and may have sustaining impacts on women postpartum. Although this current study was unable to account for adverse pregnancy outcomes, extant literature illustrates a negative association between quality of life during pregnancy and adverse pregnancy outcomes, such as unhealthy gestational weight gain, preterm birth and low birthweight (Altazan et al., 2019; Lau, 2013; Wang et al., 2013). One longitudinal study found that poor quality of life later in pregnancy was associated with preterm birth and low mental quality of life earlier in pregnancy tended to predict infants born with a low birthweight (Wang et al., 2013). It is possible that some women who had a planned caesarean section were experiencing complications during pregnancy, obesity prior to conception, or psychological challenges, as these factors are associated with lower quality of life during pregnancy (Lagadec et al., 2018; Slavin et al., 2019) and need for caesarean section (Simko et al., 2019; Solé et al., 2019). Future research is needed to explore the interrelationship between quality of life during pregnancy, mode of birth, and pregnancy outcomes and develop targeted interventions to improve quality of life among women at risk of low quality of life and adverse pregnancy and birth outcomes. It is further recommended that future research investigates the impact of pregnancy comorbidities, such as diabetes, hypertension, and preeclampsia, and other comorbidities, such as depression, on postpartum quality of life.

### Study Strengths and Limitations

This current study fills a gap in quality-of-life data for pregnant and postpartum women. Few studies have investigated the trajectory of maternal quality of life throughout pregnancy and postpartum. Previous studies have focused on quality-of-life subscale scores (e.g., physical health, mental health, social health and general health) and are limited in their applicability to other populations (Alzboon & Vural, 2019; Wang et al., 2013). We also explored both planned and unplanned caesarean section, whereas previous studies reported aggregated quality of life scores for women who had a caesarean section. Considering the push for standardized outcome reporting in maternity care, a significant strength of this study was the use of the PROMIS-10 questionnaire (Hays et al., 2009), which forms a part of the ICHOM Standard Set of Patient-centred Outcome Measures for Pregnancy and Childbirth (International Consortium for Health Outcomes Measurement, 2017). Still, the PROMIS-10 questionnaire is a generic quality-of-life instrument and lacks content validity for child-bearing women (Slavin et al., 2019), which is acknowledged as a limitation. Another limitation of this study was the recruitment of a sample from a single tertiary hospital where women are generally better educated and of higher socioeconomic status. Although the sample characteristics were broadly consistent with those observed in the Australian population, this may limit the generalisability of the findings to other healthcare settings and populations outside Australia. It is possible that the use of a utility score that combines physical and mental health quality of life may have also contributed to an underpowered study. However, the conversion of quality-of-life scores to a utility score allows for its use in future economic evaluations. Further, other confounding factors of quality of life were not included in our study due to the data availability. For example, women who report smoking in the previous 12 months or a history of mental health disorder also report lower mental health quality of life than their group counterparts, both during pregnancy and the postpartum period (Slavin et al., 2019).

## Conclusions

On average, women’s quality of life increased from 36 weeks of gestation to 12 weeks postpartum. Women who had a planned caesarean section had a significantly lower quality-of-life score prior to birth at 36 weeks of gestation compared to women who had a spontaneous vaginal birth, after adjusting for sociodemographic variables. No differences were observed after birth.

Women’s quality of life fluctuates during pregnancy and postpartum periods. Therefore, it is important to conduct quality-of-life assessments across the continuum of pregnancy and postpartum periods and consider interventions to improve the quality of life among women at higher risk of low quality of life and adverse pregnancy and birth outcomes.

## Data Availability

Our ethics approval precludes data sharing.

## Appendix 1

See Table

**Table 3:** Unadjusted mean quality-of-life utility score at 36 weeks of gestation, 6 weeks postpartum and 12 weeks postpartum, by mode of birth

	Pregnancy		Postpartum			
	36 weeks of gestation		6 weeks postpartum		12 weeks postpartum	
	Crude mean (SD)		Crude mean (SD)		Crude mean (SD)	
All women	0.82	(0.12)	0.87	(0.11)	0.88	(0.09)
	*N* = 2654		*N* = 956		*N* = 1152	
Mode of birth						
Vaginal births	0.83	(0.11)	0.87	(0.11)	0.89	(0.09)
	*N* = 1656		*N* = 605		*N* = 719	
Spontaneous vaginal birth	0.83	(0.12)	0.87	(0.11)	0.89	(0.09)
	*N* = 1396		*N* = 517		*N* = 612	
Assisted vaginal birth	0.83	(0.11)	0.87	(0.09)	0.87	(0.09)
	*N* = 260		*N* = 88		*N* = 107	
Forceps	0.81	(0.11)	0.84	(0.10)	0.84	(0.11)
	*N* = 88		*N* = 25		*N* = 33	
Vacuum	0.84	(0.10)	0.88	(0.09)	0.89	(0.08)
	*N* = 172		*N* = 63		*N* = 74	
Caesarean section	0.81	(0.12)	0.85	(0.12)	0.87	(0.10)
	*N* = 837		*N* = 295		*N* = 370	
Planned caesarean section	0.80	(0.13)	0.86	(0.12)	0.88	(0.10)
	*N* = 361		*N* = 124		*N* = 166	
Unplanned caesarean section	0.82	(0.12)	0.85	(0.14)	0.87	(0.10)
	*N* = 476		*N* = 171		*N* = 204	
Select birth type—term, spontaneous onset, vertex, no episiotomy, no epidural						
Yes	0.85	(0.10)	0.88	(0.10)	0.88	(0.10)
	*N* = 241		*N* = *241*		*N* = 241	
No	0.80	(0.13)	0.86	(0.12)	0.88	(0.10)
	*N* = 325		*N* = *325*		*N* = 325	

^3^.

## Appendix 2

See Table

**Table 4:** Coefficients across sociodemographic cohorts in the generalised linear model

Characteristics	Pregnancy			Postpartum					
	36 weeks of gestation			6 weeks of gestation			12 weeks of gestation		
	Coefficient	95% CI		Coefficient	95% CI		Coefficient	95% CI	
		Lower	Upper		Lower	Upper		Lower	Upper
Mode of birth									
Spontaneous vaginal birth	Reference								
Assisted vaginal birth	0.0070	− 0.0443	0.0592	− 0.0009	− 0.0475	0.0457	− 0.0014	− 0.0431	0.0403
Planned caesarean section	− 0.0660	− 0.1088	− 0.0243	− 0.0001	− 0.0380	0.0378	− 0.0124	− 0.0463	0.0216
Unplanned caesarean section	− 0.0153	− 0.0549	0.0244	− 0.0177	− 0.0532	0.0178	− 0.0156	− 0.0475	0.0162
Age (years)									
< 20	Reference								
20–24	− 0.0104	− 0.3367	0.3158	− 0.1246	− 0.4182	0.1690	− 0.1346	− 0.3974	0.1282
25–29	0.0080	− 0.3151	0.3311	− 0.1318	− 0.4227	0.1591	− 0.1331	− 0.3933	0.1271
30–34	0.0033	− 0.3196	0.3261	− 0.1372	− 0.4279	0.1535	− 0.1412	− 0.4012	0.1189
35–39	0.0023	− 0.3200	0.3247	− 0.1476	− 0.4378	0.1426	− 0.1302	− 0.3899	0.1295
40 +	0.0317	− 0.2952	0.3586	− 0.1684	− 0.4625	0.1257	− 0.1321	− 0.3953	0.1310
Education									
Less than year 12	− 0.0299	− 0.0898	0.0299	− 0.0005	− 0.0545	0.0534	− 0.0110	− 0.0593	0.0373
Secondary school	− 0.0097	− 0.0533	0.0339	− 0.0287	− 0.0679	0.0105	− 0.0054	− 0.0405	0.0297
Apprenticeship or diploma	− 0.0374	− 0.0707	− 0.0041	− 0.0364	− 0.0663	− 0.0064	− 0.034	− 0.0608	− 0.0072
University/tertiary degree	Reference								
Socioeconomic status (SEIFA)									
1 (the most disadvantaged)	Reference								
2	0.0234	− 0.0333	0.0801	0.0134	− 0.0376	0.0644	0.0053	− 0.0402	0.0509
3	0.0099	− 0.0421	0.0618	− 0.0047	− 0.0514	0.0421	− 0.0046	− 0.0464	0.0371
4 (the least disadvantaged)	0.0214	− 0.0329	0.0756	− 0.0157	− 0.0646	0.0331	− 0.0239	− 0.0675	0.0198
Parity									
Parous	− 0.0012	− 0.0323	0.0298	0.0184	− 0.0094	0.0463	0.0117	− 0.0132	0.0367
Nulliparous	Reference								
Pre-pregnancy body mass index (kg/m^2^)									
Underweight (< 18.5)	0.0030	− 0.0757	0.0817	0.0207	− 0.0503	0.0916	− 0.0031	− 0.0666	0.0603
Normal weight (18.5–24.9)	Reference								
Overweight (25.0–29.9)	− 0.0254	− 0.0595	0.0086	− 0.0100	− 0.0407	0.0206	− 0.0137	− 0.0411	0.0137
Obese (≥ 30.0)	− 0.0468	− 0.084	− 0.0095	− 0.0370	− 0.0704	− 0.0035	− 0.0276	− 0.0577	0.0024
Marital status									
Single	− 0.0445	− 0.1118	0.0227	− 0.0220	− 0.0826	0.0386	− 0.0163	− 0.0704	0.0378
Married/de Facto/ domestic partner	Reference								
Country of birth									
Australia	Reference								
Indigenous Australian	− 0.0148	− 0.0943	0.0646	− 0.0750	− 0.1465	− 0.0035	− 0.0439	− 0.1081	0.0202
Asian	− 0.0250	− 0.1180	0.0679	− 0.0067	− 0.0905	0.077	− 0.0349	− 0.1098	0.0400
New Zealand	0.0057	− 0.0457	0.0571	0.0514	0.0052	0.0976	0.0157	− 0.0256	0.0571
Other	0.0105	− 0.0298	0.0508	0.0065	− 0.0298	0.0428	− 0.0288	− 0.0613	0.0037

*CI* confidence interval

4.

## Code Availability

Available upon request.
